# Student Perceptions of Assessment for Workplace Learning and Summative Decisions Using Frequent Mini-CEXs

**DOI:** 10.15694/mep.2017.000113

**Published:** 2017-06-22

**Authors:** Justin J. Mowchun, Mary S. Feldman, Glenda Hostetter Shoop

**Affiliations:** 1 Geisel School of Medicine at Dartmouth; 2 Wright State University Boonshoft School of Medicine; 3 Geisel School of Medicine at Dartmouth

**Keywords:** assessment, feedback, mini-CEX, summative, workplace-based

## Abstract

This article was migrated. The article was marked as recommended.

**Introduction**: Direct observation assessments that provide both formative feedback and data for summative decisions can be difficult to achieve. The mini-clinical evaluation exercise (mini-CEX) is a widely used tool of direct observation that provides opportunities for feedback. We introduced a direct observation system with frequent mini-CEXs to increase clerkship student learning opportunities and to improve competency-based summative decisions. However, students may express resistance to assessments for learning with any summative impact as they may perceive the assessments as purely a series of summative evaluations.

**Aims**: We explored how frequent low-stakes mini-CEXs affect clerkship students’ perception of learning and to understand student perceptions of these assessments supporting their end-of-rotation summative clinical performance evaluations.

**Methods**: This qualitative study used a purposive sampling strategy of focus groups with students who completed multiple mini-CEXs during their four-week neurology clerkship at one of three sites. All eleven students chose to participate. Eight students completed eight mini-CEXs, two students completed seven, and one student completed four. Investigator triangulation was used with interpretation comparisons that included independent content analysis. The emerging themes were discussed and final theme consensus was reached.

**Results**: Three major themes arose: perceived effects of frequent mini-CEXs for clerkship student learning by facilitating multiple opportunities for guided practice under low assessment anxiety; the importance of consistent, effective faculty feedback and engagement to maximize mini-CEXs for learning; and support for summative impact of frequent, mainly formative, low stakes mini-CEXs.

**Conclusions**: Students perceived that frequent mini-CEXs are mainly formative assessments for learning while having summative impact. However, variable perceived faculty affinity and engagement with the mini-CEX are important considerations to maximize the assessments for learning. These findings support a shift towards workplace-based assessment programs for learning that promote frequent direct observation and feedback, while also improving the trustworthiness of summative decisions.

## Introduction

The move towards competency-based medical student clinical education requires advancement in assessment approaches ( Epstein & Hundert, 2002). Assessment of workplace-based clinical performance is emphasized in clerkships. This clinical environment intends to evaluate the student at the “does” level; in the real-life setting as opposed to a simulated context ( Miller, 1990). However, assessment of authentic workplace-based clinical performance poses significant challenges. It is important to recognize the limitations of isolated assessment data points ( Van der Vleuten et al., 2012). All assessment methods require extensive sampling, and workplace-based performance assessments are no different. The main factor affecting the reliability of these assessments is content-specificity because competence is highly dependent on context (Van der Vleuten, 1996). One way to achieve reliable measurements of medical student clinical competence is to assess a large sample across the clerkship clinical experience.

Another important consideration of an isolated assessment point is that it will not be able to facilitate and determine student growth. The limitation of single data points of assessment can help reframe thinking about programs of assessment ( Van der Vleuten et al., 2012). Assessment for learning is a more recently described view of assessment in medical education. It is an approach in which the assessment process is inextricably embedded within the educational process that is information-rich, and guides the learning of each student to the maximum of their ability ( Schuwirth & Van der Vleuten, 2011). In this framework, it has been argued that for an assessment to be meaningful and informative for learning it requires some “teeth”; the formative and summative function of assessment should be combined when possible ( Shuwirth & Ash, 2013). High-stakes decision making is informed by many data points and low-stakes assessment should feed into high-stakes information. It is important for even very low-stakes assessment to carry some summative impact. However, it is commonly believed that an assessment cannot fulfil the dual purpose of offering summative measurement and formative guidance. This approach can be detrimental as it risks absolving the training programs that are responsible for guiding learners from serving as effective gate keepers ( Eva et al., 2016).

Global clinical performance ratings are the dominate method for summative evaluations of clerkship clinical competency ( Kassebaum & Eaglen, 1999 *).* The strengths of these evaluations are that they reflect longitudinal student performance in the real clinical care context, and require minimal time beyond the day-to-day workplace interactions of student and faculty ( Holmboe & Hawkins, 2008). The problems with these evaluations are well known. Assessment of performance over one to several weeks places high demand on memory of specific observations. The process of summarizing these events from memory into an average level for a specific competency domain opens up opportunities for bias ( Albanese, 2000). Workplace-based student performance may be overestimated and global evaluations are often weighted towards presentation skills ( Norcini et al., 2003). The culture of a summative evaluation, where students focus on grades, acts as an inhibitor of feedback utilization for learning ( Harrison et al., 2015). These evaluations are also unable to provide timely feedback to reinforce good clinical skills from direct observation of patient encounters, and help identify and correct performance deficiencies.

It is well known that direct observation and evaluation of medical trainee’s patient encounters are integral for clinical skills competency assessment ( Holmboe, 2015). Direct observation can also facilitate deliberate practice, which involves repetitive performance of specific cognitive and psychomotor skills in the context of rigorous assessment (including self-assessment), with timely informative feedback to achieve a higher level of performance ( Erickson, 2015). The mini clinical evaluation exercise (mini-CEX) is a widely used and studied tool of direct observation assessment of clinical skills that provides opportunities for feedback ( Norcini & Burch, 2007). It has been reported that high reliability of the mini-CEX can be achieved with a sample of 8 to 10 encounters (Pelgrem et al., 2011). The literature suggests that multiple mini-CEX measurements are valid for resident and medical student performance ( Kogan et al., 2009). Published reports of its valid frequent use for medical students include an internal medicine clerkship where students were instructed to participate in 10 mini-CEXs during a 9-week rotation ( Kogan et al., 2003). This report emphasized that the mini-CEX was used in a purely formative assessment role; however, student perceptions of mini-CEX educational impact were not obtained. The mini-CEX psychometric properties also indicate that it could be used for summative purposes ( Alves de Lima et al., 2007). It is unclear if frequent mini-CEXs can be formative assessments that guide clerkship student learning, but with summative impact as well.

Clerkship students may express resistance to assessments for learning with any summative impact as they may perceive the assessments as purely a series of high-stakes summative evaluations. Negative pre-assessment effects on medical student learning are well known and include predominate study activity right before the assessment or cue seeking ( Cilliers et al., 2012). Clinical workplace-based assessments that provide meaningful feedback and data for summative decisions are very difficult to achieve, as students perceive low-stakes formative assessments as summative ( Bok et al., 2013). Bok et al proposed that successful implementation may be conditional on specific faculty and student feedback and assessment program training. However they concluded that further research should focus on whether, and in which conditions, low-stakes formative assessments are perceived as low-stakes for learning. The credibility of the feedback giver is also important to medical traineesfor learning ( Dijksterhuis et al., 2013). The mini-CEX and other workplace-based assessments are perceived as “tick box” exercises for many trainees instead of learning opportunities ( Bindal et al., 2011; Weston et al., 2014). A study of preclinical second year medical students also concluded that assessment for learning activities support or inhibit learning based on how these activities are perceived by the individual learner ( Heeneman et al., 2015). When these learners understood an assessment purpose, they would more likely buy into it. However, they perceived low-stakes formative assessments as high-stakes and purely summative.

A study was performed to gain insight into the following research questions: (1) How do frequent low-stakes mini CEX’s affect clerkship students’ perception of learning? (2) What are clerkship students’ perceptions of these low-stakes, mainly formative assessments, supporting their end-of-rotation summative clinical performance evaluations?

## Methods

### Setting

The study was conducted at Dartmouth Hitchcock Medical Center after a major reorganization of the neurology clerkship’s direct observation assessment system, which included a modified mini-CEX form (available under appendicies). The mini-CEX behavioral anchors aligned with the end-of-rotation performance evaluation anchors. Previously, the clerkship required one formative direct observation of each student’s complete neurological exam with a checklist. The new system was introduced February 29, 2016, which was in the final quarter of the academic year. Nineteen neurology attendings and two chief residents were oriented to the mini-CEX assessment for learning system with JM. This orientation occurred in small groups or individually for about 40 minutes, within one month of their first opportunity to complete a mini-CEX. Feedback training was also included in the orientation using the Ask-Tell-Ask Model to promote learner reflection and self-assessment ( French et al., 2015).

Students were oriented to the system as part of their clerkship orientation session with JM. A student feedback framework was discussed to emphasize the value of learner self-assessment and receptivity of specific mini-CEX feedback for learning. Empowering students with a process on how to receive and handle specific feedback, regardless of the way it is given, can improve feedback in the busy clinical workplace setting ( Algiraigri, 2014).

Frequent direct observation assessments occurred during the four-week clerkship. Students were asked to complete eight mini-CEXs (at least one to three per week) to promote student learning and evaluate clinical competence. Students were assessed by at least three faculty over their clerkship. The assessments took place in the inpatient and outpatient setting. The students kept their completed mini-CEXs, and at the end of the clerkship delivered their forms to the clerkship administrator who created a mini-CEX file as a robust data set on each student’s clinical performance. All mini-CEX and global performance evaluations were sent to the clerkship education committee at the end of the rotation. Expert committee judgment is needed for aggregating the information across all data points to evaluate clinical competence ( Van der Vleuten et al., 2012). The mini-CEX data was triangulated with the global performance evaluation data; combining assessment data on context similarity across methods is more meaningful for a competency-based, assessment for learning program ( Shuwirth & Ash, 2013). To remain consistent across the current academic year, the weight of global performance evaluations for final grade determination did not change. However, the students were informed that the mini-CEX data would be used qualitatively to support their global performance evaluations for the competencies of patient care, communication, and professionalism, and could impact the committee’s final summative decision. In the complex environment of the clinical workplace, competency systems that are grounded with a qualitative research paradigm may be an effective, fair, traceable, and a highly defensible approach ( Govaerts & van der Vleuten, 2013).

### Ethical Considerations

This study was given exemption status by the Committee for the Protection of Human Subjects (Dartmouth College/ Dartmouth Hitchcock Medical Center). Potential participants were informed about the voluntary nature of the study and that data would be audio recorded and analyzed anonymously. They were also informed that data would be analyzed after their final grades were submitted.

### Sampling

The study used a purposive sampling strategy of focus group sessions with third and fourth year medical students who had been oriented to the new direct observation system, and completed multiple mini-CEXs during their neurology clerkship. All eleven of these students (6 male, 5 female) chose to participate in the focus groups. Eight students completed eight mini CEXs, while two students completed seven, and one student completed four. The majority of students completed their clerkship at Dartmouth-Hitchcock Medical Center in Lebanon, New Hampshire, although three students completed their clerkship at a nearby clerkship site (Veterans Administration Medical Center in White River Junction, Vermont). One student completed the clerkship at a Dartmouth-Hitchcock affiliated site in Manchester, New Hampshire. The focus groups took place at Dartmouth-Hitchcock Medical Center on the final day of the clerkship. The first focus group had three participants and the second focus group, one month later, had eight participants. The focus group sizes were dependent on variable clerkship enrollment for each block. The optimal size of a focus group has been reported to be between six to ten participants ( Barbour, 2005). However, a minimum of three to four participants is possible for a focus group ( Stalmeijer et al., 2014).

### Data collection

Focus groups were utilized to facilitate interaction between the students to gain depth in the exploration of the topics, make interconnections, and understand student interactions on the topics ( Parker & Tritter, 2006). The moderator (GHS) had background knowledge of the clerkship but was not involved in student evaluation. Semi-structured interview questions were created based on work in the areas of workplace clinical assessment for learning, performance feedback, and clinical competency ( Bok et al., 2013; Dijksterhuis, et al., 2013; Weston et al., 2014). The moderator facilitated the discussion using a list of questions for guidance ( Table 1).

### Data Analysis

The focus groups were audio recorded and verbatim transcripts were created. Each focus group was analyzed after final student grades were submitted. Focus groups and analyses were conducted iteratively to facilitate the expansion of categories in the second focus group. Focus group data was compared within each group and also among groups ( Kruger, 1997). A phenomenological approach to data interpretation was used as these medical students had their own unique experiences ( Crabtree & Miller, 1999). Investigator triangulation was used with interpretation comparisons among JM and MF that included independent content analysis, and major themes emerged after extensive discussion of the codes. The emerging themes were discussed and reviewed with GHS and final theme consensus was reached. Data saturation was deemed to be achieved after the second focus group, as no new ideas about the topic emerged. Member checking with the students was not performed, as this process can lead to confusion rather than confirmation. Participants may later change their mind about their perspective, and new clerkship experiences since the focus groups could have intervened ( Angen, 2000).

**Table 1.**
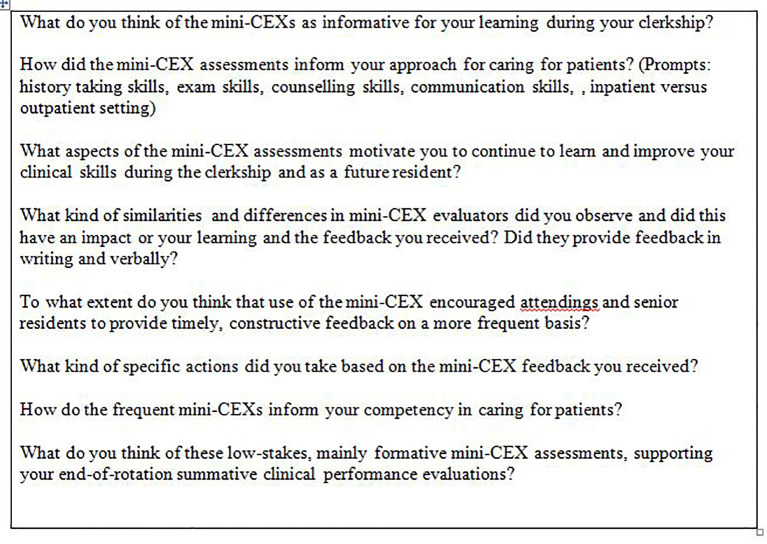
Questions: focus group clerkship students

## Results

Three major themes arose from the focus groups: perceived effects of frequent mini-CEXs on clerkship student learning, consistent effective faculty feedback and engagement to maximize perception of mini-CEX assessments for learning, and support for summative impact of frequent, mainly formative, low-stakes mini-CEXs. We will present the results, supported by distinctive quotes from both focus groups, organized according to themes below and in Table 2.

### Perceived effects of frequent mini-CEXs on clerkship student learning

Students perceived that frequent mini-CEXs facilitated a more comfortable assessment environment for learning and avoided assessment anxiety.

..doing eight of them, eventually, they become less of a big deal for the student. I mean, it feels like if you have one of these things to do on a clerkship, it feels like more of a big deal but if there’s eight of them, it’s kind of less weight to each of them so that’s good. It becomes part of the flow more.

Students described a process of guided practice that took advantage of multiple assessment moments as authentic learning moments.

..even though like there were so many times I did exams that were not part of a mini-CEX but when they were part of it, before I went in I was like, Okay, this is how I want to do it. I want to do it in this order. Oh, I don’t want to forget that thing that I forgot last time. So it was more just slightly more hyper aware of like being prepared for like a formal exam and wanting to do it in order without skipping around and forgetting a few things. So that was nice because then I felt like by the end, I was more prone to just keep doing it that way and be like hyper aware every time..

### Consistent, effective faculty feedback and engagement to maximize perception of mini-CEX assessment for learning

Students emphasized that frequent timely feedback was not sufficient when the quality of the feedback between evaluators was inconsistent. Each feedback encounter must be perceived as high value to maximize the mini-CEX assessments for learning.

..I just think it’s so dependent on the feedback that you get and the willingness of the person to kind of try to give you a pearl of some sort that you can go from, you know, move forward with.

Students valued constructive feedback with the mini-CEX and guidance with a focused action plan so they could improve.

..So for counseling..I ended up using medical jargon and I tried to catch myself at times but in the end, like he’d come to like, “Oh, yeah, I can see that you’re sort of struggling with trying to modify your explanation to the level of the patient.. it influenced the way I have approached patients subsequently for that.

However, students also perceived that inconsistent assessor engagement and feedback effectiveness limited the mini-CEX assessment for learning process.

..I felt like people that did it well, you know, included specific feedback, things that you could really hang your hat on and go with. Whereas, you know, the people who did it poorly where it was just kind of vague encouragement as feedback, which didn’t really go very far.

..it’s so dependent on the teacher, basically. And some people are great teachers and they’re going to be great teachers and they’re going to take the time to do that stuff. And some people..that’s a lower priority or they do it in different ways.

A minority of students who described being frequently observed by attendings as part of work flow in one mainly outpatient site did not find that the formal mini-CEX component improved the feedback they received, in the context of perceived lower faculty engagement with the mini-CEX form.

..Over the course of that day, they will watch you do ten exams back to back. They give you feedback as you go. Usually we just found some time to fill these out afterward.

However, at another mainly outpatient site, the faculty engagement with the mini-CEX process was perceived as very high and facilitated completion of frequent mini-CEXs.

..I had a different experience. The attendings ...who do them are 100% on board with doing them. They would ask me for them. It makes it way easier.

Even though students emphasized that some of their mini-CEX encounters ended with unsatisfying feedback for learning, they did perceive the mini-CEX augmented the potential for more frequent specific feedback discussions while they took care of patients on their clerkship. They recognized that the frequent mini-CEXs increased clerkship expectations of regular performance feedback for learning and improvement.

..I think more than my other inpatient rotations, I got more feedback directly back from attendings on this clerkship because of this exercise. Usually, when we were on an inpatient team with the residents and interns - the attending doesn’t really see the medical student do things very often..

### Support for summative impact of frequent, mainly formative, low-stakes mini-CEXs

The mini-CEXs were perceived as assessments for learning and not a series of summative assessments. The majority of student’s mini-CEXs were done with attendings and chief residents that would be filling out an end-of-rotation clinical performance evaluation. Students supported the use of formative mini-CEX data into these evaluations.

..I think it is helpful because it forces them to have to see you do something, as opposed to judging you on whatever they usually judge you on.

Although the students did perceive the mini-CEXs as primarily for their learning, they were also generally accepting of the assessments minimal summative impact as part of their mini-CEX file supporting their end of rotation global performance evaluations.

..Okay, I think I’m doing alright in Neurology. Then, you know, having a little piece of paper that says, “No, really, you’re doing alright,” .. just reassurances, I guess.

However, the majority of students emphasized the importance of faculty completing end-of-rotation global performance evaluations as the dominant summative work-place based assessment.

..The one reason I like the current system is that you can almost kind of forget about it. You know every day you want to try to do really well..You can also forget about the grading aspect a little bit.

**Figure:**
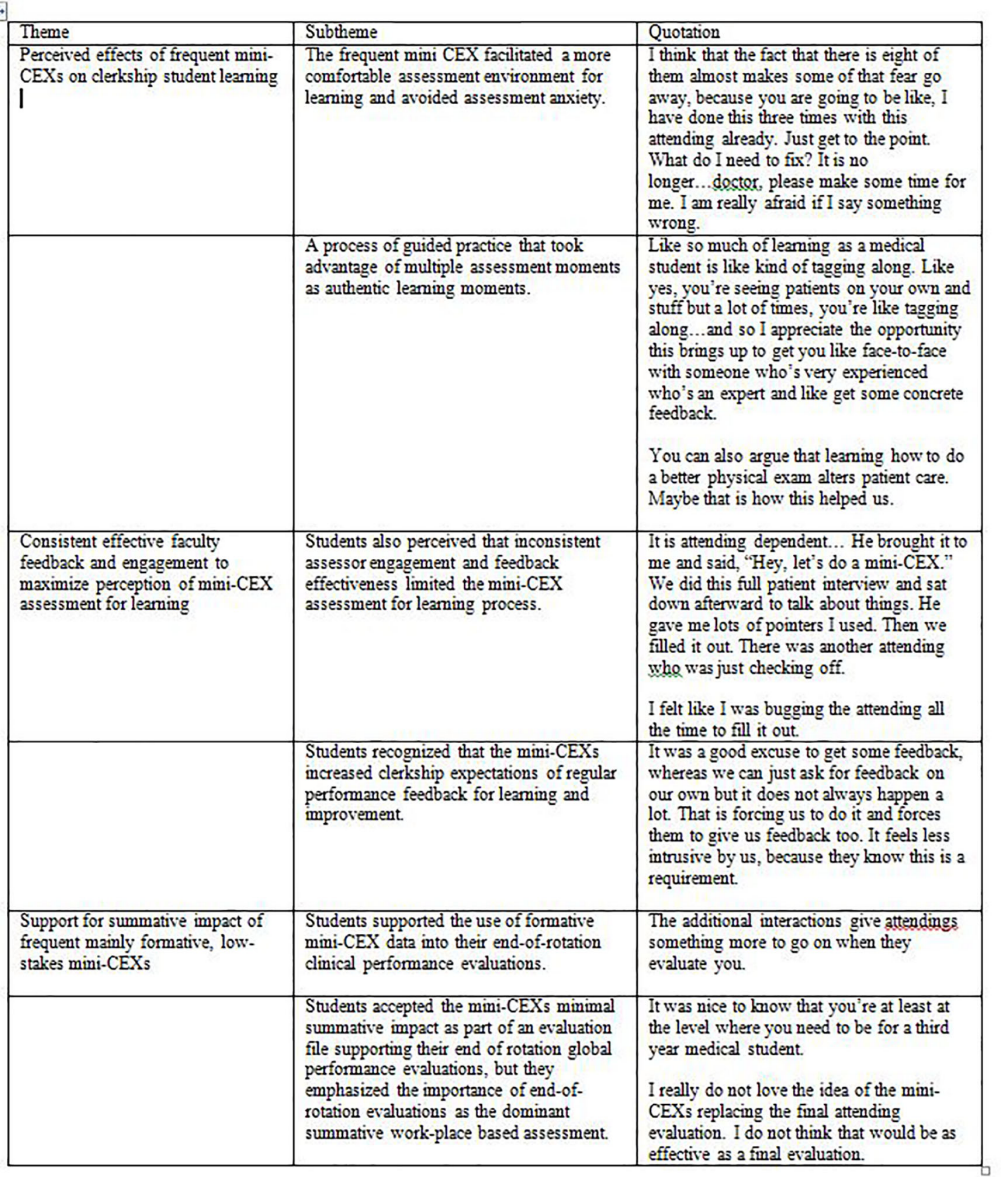


## Discussion

The students endorsed that frequent mini-CEXs facilitated a workplace environment for guided learning. From the clinical workplace literature, education tends to be perceived as embedded in day-to-day clinical work, with effective patient care the primary motivator. Physicians and physician trainees do not tend to engage in the deliberate practice that many other professionals undertake to improve and achieve a high level of competency ( van de Wiel et al., 2011). However, our clerkship students recognized the educational value of the mini-CEX assessments as a form of deliberate practice, which is more than just repeatedly examining and evaluating their patients. The students perceived effective learning, under the watchful eyes of experienced physicians to improve and enhance their clinical skills.

The frequent mini-CEXs also facilitated a student culture of deliberate practice for clinical learning during their clerkship. Students’ active engagement in the deliberate practice process is essential for the acquisition and improvement of clinical skills via this method ( Duvivier et al., 2011). Our students perceived that the frequency of the mini-CEXs reduced the stakes of each assessment and thereby facilitated an environment for learning. Previous work with infrequent formative mini-CEXs for medical residents, even without any summative impact, still led to significant anxiety having their performance observed and rated; this led to significant limitations of its perceived value as a learning experience ( Malhortra et al., 2008). However, a minority of these residents who completed more mini-CEXs seemed more open to it as a learning opportunity, and the study suggested that these learners were more confident in controlling the positive educational effects of the mini-CEX.

The students also generally valued the frequent mini-CEX opportunities to receive timely feedback on their performance. Although it is unclear how often feedback should be given during deliberate practice in the clinical environment, a recent randomized controlled trial concluded that medical student procedural skills performance via deliberate practice was greater with high frequency intermittent feedback compared to low frequency intermittent feedback ( Bosse et al., 2015). The mini-CEX does not assess procedural skills, however clinical skills performance may also benefit from a high frequency feedback approach.

It is important to recognize that more frequent timely feedback is insufficient for students to realize the potential of the mini-CEX for learning. Each feedback encounter must be of high value to fully maximize the mini-CEX assessments for learning. Even with focused timely faculty feedback training, students perceived that some faculty had difficulty discussing specific meaningful areas for them to improve and a subsequent action plan. This is consistent with a study that found inconsistent documentation of constructive feedback to medical students with the mini-CEX ( Fernando et al., 2008). Action plans in that study were even more difficult for faculty to suggest, as in 50% of cases no action plan was developed. However, constructive feedback with a specific measurable action plan for improvement is essential for each mini-CEX to be an effective assessment for learning, and our findings also reflected this inconsistent component of our feedback process. Perceptions of consistent high quality feedback with the mini-CEX were also found to be difficult to obtain in other studies with students ( Bok et al., 2013) and residents ( Bindal et al., 2011; Malhotra et al., 2008).

Students were more supportive of the potential of the mini-CEX feedback process for learning, possibly because guidance and scaffolding on receiving feedback with the mini-CEX was emphasized during their clerkship orientation. Students had also done infrequent purely formative direct observation assessment in other clerkships. Scaffolding provides student direction, motivation, and goal definition of the feedback process ( Archer, 2010). However, a more narrow view of effective feedback being timely and specific may be insufficient for assessment for learning, even with students who are open to receive it. There is an argument to focus more on the teacher-learner relationship for effective feedback ( Bing-You & Trowbridge, 2009). Faculty may avoid giving constructive feedback to students due to concerns that the feedback may damage their relationship and that a lower performance evaluation may impair student motivation ( Daelmans et al., 2006). Also, the mini-CEX is a form of face-to-face performance feedback and previous work concluded that faculty are poor at giving direct, specific, face-to-face feedback, especially when it involves areas for the student to improve ( Colletti, 2000). A residency program discovered that faculty identified giving constructive feedback as a very difficult personal challenge and had difficulty doing it without perceived harm to their residents ( Watling et al., 2010). Measurable and specific action plan development has also been reported as difficult to implement, as faculty may be uncomfortable with the process ( Holmboe et al., 2004).

Students emphasized that some faculty were able to facilitate positive and constructive feedback with an action plan that exemplified assessment for learning with the mini-CEX. Students were also appreciative of many expert faculty (chief residents and attending) who directly observed them as part of a mini-CEX in the busy clinical workplace, and that expertise was perceived as an important component of feedback credibility. However, this aspect of credibility was not sufficient for optimal assessment for learning. Respect for expert evaluators has been shown to be important for feedback credibility, however the content of the feedback and method of feedback delivery were also important for credibility ( Bing-You et al., 1997 **)**. Another study found that residents’ perception of joint engagement of assessor and learner to the evaluation and feedback process is essential ( Watling et al., 2008). Assessor engagement and commitment also influence learner perceptions of assessor credibility along with feedback being received in a supportive learning environment ( Dijksterhuis et al., 2013) . The meaningful use of a workplace assessment innovation is also dependent on the user’s affinity with the assessment instruments ( Fokkema et al., 2013). Our students perceived that there was variable faculty affinity with the mini-CEX, which for some students affected the ease of completing multiple mini-CEXs, and affected the quality of some of their mini-CEXs for learning.

Our clerkship has been able to jump start an assessment for learning culture that better recognizes the importance of regular direct observation and feedback for student learning. However, to further promote a culture of feedback it will be essential for faculty to continue to work together as an organization that values feedback skills. This work includes the recognition of the difficult emotional and affective aspects of the feedback process for assessor and learner ( Bing-You & Trowbridge, 2009). Frequent mini-CEXs help an organization’s system to increase expectations for student learning and help better monitor the feedback process in a timely manner ( Ramani & Krackov, 2012). Faculty should consider giving feedback after direct observations as a skill that can be developed and improved through repeated practice ( Cantillon & Sargeant, 2008). Another consideration for faculty is incorporating a more formalized approach to acknowledging the emotional aspect of feedback prior to exploring specific content, as outlined by a framework used with some success in the multisource feedback environment ( Sargeant et al., 2011). It is important to routinely discuss assessment successes and problems with faculty to continue to improve affinity with the mini-CEX. To achieve and maintain user readiness of innovation, users need to experiment, challenge, evaluate, and find meaning in the innovation ( Greenhalgh et al., 2004). It is also important to continue to analyze the complex system dynamics of the clinical and education service of an organization to realize innovation ( van Rossum et al., 2016).

Our data analysis concludes that students perceived that frequent mini-CEXs were formative assessments for their learning, but they also accepted that the mini-CEXs carried minimal summative impact. The students did not perceive the mini-CEX as a series of summative assessments. This is in contrast to other studies in the clinical workplace where perception of summative impact of the mini-CEX prevailed ( Bindal et al., 2011; Bok et al., 2013). This problem has also been well described in an undergraduate pre-clinical curriculum assessment program where students did not regard formative assessments as such and instead interpreted them as summative assessment activities where pre-assessment effects dominated ( Heeneman et al., 2015). Our students did not endorse the common and often negative pre-assessment effects that include attempts to memorize just prior to the assessment or cue seeking ( Cilliers et al., 2012). One possible explanation for this finding is that timely student and faculty development of the direct observation system may help support the integrity of the workplace assessment for learning ( Fokkema et al., 2013). It is important to note that if the assessor and assesse do not fully understand the meaning and purpose of the assessment it will be trivialized, which is a common and serious problem in assessment ( van der Vleuten et al., 2012). The students were oriented to the new system on the first day of their clerkship and faculty were oriented in small groups or individually within one month of their first opportunity to do a mini-CEX. It is also important to recognize that since the students’ end-of-rotation global performance evaluations were the primary focus of their summative competency evaluation (along with the National Board of Medical Examiners (NBME) subject exam), the students may have been more open to the integration of frequent mini-CEXs as a low-stakes component of their summative evaluation. The question is not whether an assessment is mainly summative or formative but the degree an assessment’s formative and summative purposes are focused in the mind of the learner ( Eva et al., 2016). End- of- rotation clinical performance evaluations may help facilitate student perception that frequent low-stakes mini-CEXs are mainly for their learning, and avoid the common perception that the assessments are only a series of summative evaluations. In contrast, faculty preceptors did not complete end-of-rotation clinical performance evaluations in the study by Bok et al, ^23^.

### Limitations

Limitations of this study include that the sample of students is from one United States medical school; however students who participated completed their clerkship at three different sites. The study only focused on student perceptions of one type of direct observation workplace-based assessment. The study also only sought student perceptions; faculty assessor perceptions of the assessment system would also be important to understand. Previous work suggests that any examination of perceptions in the clinical workplace assessment process is incomplete unless the perceptions of the assessors are also considered (Watling et al., 2012). JM and MF had direct although limited contact with many of the students through the clerkship, which carried potential reflexive effects. However, the focus groups were carried out by GHS who was not directly involved with the clerkship.

## Conclusion

The current findings contribute to the research evidence of clinical workplace-based assessment for learning. Frequent mini-CEXs can be mainly formative assessments to facilitate student learning while having summative impact. Students perceive that frequent mini-CEXs reduce the stakes of each assessment and facilitate an environment for learning. End-of rotation clinical performance evaluations may help reinforce the mainly formative purpose of frequent mini-CEXs in the student’s mind. Our findings also emphasize the complexities and challenges of the dynamic assessment and feedback process in the clerkship workplace. These findings may give clerkships a springboard to become assessment programs for learning, which reflect a culture of frequent direct observation and feedback of student trainees. At the same time these changes can improve the trustworthiness of summative evaluation decisions for each student.

## Take Home Messages

• Students perceive that frequent mini-CEXs reduce the stakes of each assessment and facilitate an environment for learning.
• Despite timely faculty training, students perceive that inconsistent assessor engagement and feedback effectiveness can limit the educational value of a mini-CEX.
• Frequent mini-CEXs can be perceived as assessments for learning with summative impact when they are used to support student global performance evaluations.

## Notes On Contributors

Justin Mowchun MD,MScEd is an Assistant Professor of Neurology and is the Director of the Neurology Clerkship, Geisel School of Medicine at Dartmouth, Dartmouth Hitchcock Medical Center, Lebanon, New Hampshire.

Mary Feldman DO is an Assistant Professor of Neurology, Wright State University Boonshoft School of Medicine, Premier Health Clinical Neuroscience Institute, Dayton, Ohio. Dr. Feldman was an Assistant Professor of Neurology, Geisel School of Medicine at Dartmouth, at the time of the study.

Glenda Hostetter Shoop PhD is the Director of Curriculum Design and Evaluation, Geisel School of Medicine at Dartmouth, Lebanon, New Hampshire.

## References

[ref1] Albanese M. A. (2000). Challenges in using rater judgements in medical education. Journal of Evaluation in Clinical Practice. 6(3),305–319. 10.1046/j.1365-2753.2000.00253.x 11083041

[ref2] Algiraigri A. H. (2014). Ten tips for receiving feedback effectively in clinical practice. Medical Education Online. 19,25141. 10.3402/meo.v19.25141 25079664 PMC4116619

[ref3] Alves de Lima A. Barrero C. Baratta S. Castillo Costa Y. Bortman G. Carabajales J. Van der Vleuten C. (2007). Validity, reliability, feasibility and satisfaction of the mini-clinical evaluation exercise (mini-CEX) for cardiology residency training. Medical Teacher. 29(8),785–790. 10.1080/01421590701352261 17917984

[ref4] Angen M. J. (2000). Evaluating interpretive inquiry: Reviewing the validity debate and opening the dialogue. Qualitative Health Research. 10(3),378–395. 10.1177/104973230001000308 10947483

[ref5] Archer J. C. (2010). State of the science in health professional education: Effective feedback. Medical Education. 44(1),101–108. 10.1111/j.1365-2923.2009.03546.x 20078761

[ref6] Barbour R. S. (2005). Making sense of focus groups. Medical Education. 39(7),742–750. 10.1111/j.1365-2929.2005.02200.x 15960795

[ref7] Bindal T. Wall D. & Goodyear H. M. (2011). Trainee doctors’ views on workplace-based assessments: Are they just a tick box exercise? Medical Teacher. 33(11),919–927. 10.3109/0142159X.2011.558140 22022902

[ref8] Bing-You R. Paterson J. & Levine M. A. (1997). Feedback falling on deaf ears: Residents’ receptivity to feedback tempered by sender credibility. Medical Teacher. 19(1),40–44. 10.3109/01421599709019346

[ref9] Bing-You R. G. & Trowbridge R. L. (2009). Why medical educators may be failing at feedback. Jama. 302(12),1330–1331. 10.1001/jama.2009.1393 19773569

[ref10] Bok H. G. Teunissen P. W. Favier R. P. Rietbroek N. J. Theyse L. F. Brommer H., . . . Jaarsma D. A. (2013). Programmatic assessment of competency-based workplace learning: When theory meets practice. BMC Medical Education. 13, 123-6920-13-123. 10.1186/1472-6920-13-123 PMC3851012 24020944

[ref11] Bosse H. M. Mohr J. Buss B. Krautter M. Weyrich P. Herzog W. Nikendei C. (2015). The benefit of repetitive skills training and frequency of expert feedback in the early acquisition of procedural skills. BMC Medical Education. 15, 22-015-0286-5. 10.1186/s12909-015-0286-5 PMC4339240 25889459

[ref12] Cantillon P. & Sargeant J. (2008). Giving feedback in clinical settings. BMJ (Clinical Research Ed.). 337,a1961. 10.1136/bmj.a1961 19001006

[ref13] Cilliers F. J. Schuwirth L. W. Herman N. Adendorff H. J. & van der Vleuten C. P. (2012). A model of the pre-assessment learning effects of summative assessment in medical education. Advances in Health Sciences Education: Theory and Practice. 17(1),39–53. 10.1007/s10459-011-9292-5 21461880 PMC3274672

[ref14] Colletti L. M. (2000). Difficulty with negative feedback: Face-to-face evaluation of junior medical student clinical performance results in grade inflation. The Journal of Surgical Research. 90(1),82–87. 10.1006/jsre.2000.5848 10781379

[ref15] Crabtree B. F. & Miller W. L. (1999). Doing qualitative research.( 2nd Ed.). Thousand Oaks, CA: Sage.

[ref16] Daelmans H. E. Overmeer R. M. van der Hem-Stokroos H. H. Scherpbier A. J. Stehouwer C. D. & van der Vleuten C. P. (2006). In-training assessment: Qualitative study of effects on supervision and feedback in an undergraduate clinical rotation. Medical Education. 40(1),51–58. 10.1111/j.1365-2929.2005.02358.x 16441323

[ref17] Dijksterhuis M. G. Schuwirth L. W. Braat D. D. Teunissen P. W. & Scheele F. (2013). A qualitative study on trainees’ and supervisors’ perceptions of assessment for learning in postgraduate medical education. Medical Teacher. 35(8),e1396–402. 10.3109/0142159X.2012.756576 23600668

[ref18] Duvivier R. J. van Dalen J. Muijtjens A. M. Moulaert V. R. van der Vleuten C. P. & Scherpbier A. J. (2011). The role of deliberate practice in the acquisition of clinical skills. BMC Medical Education. 11, 101-6920-11-101. 10.1186/1472-6920-11-101 PMC3293754 22141427

[ref19] Epstein R. M. & Hundert E. M. (2002). Defining and assessing professional competence. Jama. 287(2),226–235. 10.1001/jama.287.2.226 11779266

[ref20] Ericsson K. A. (2015). Acquisition and maintenance of medical expertise: A perspective from the expert-performance approach with deliberate practice. Academic Medicine: Journal of the Association of American Medical Colleges. 90(11),1471–1486. 10.1097/ACM.0000000000000939 26375267

[ref21] Eva K. W. Bordage G. Campbell C. Galbraith R. Ginsburg S. Holmboe E. & Regehr G. (2016). Towards a program of assessment for health professionals: From training into practice. Advances in Health Sciences Education: Theory and Practice. 21(4),897–913. 10.1007/s10459-015-9653-6 26590984

[ref22] Fernando N. Cleland J. McKenzie H. & Cassar K. (2008). Identifying the factors that determine feedback given to undergraduate medical students following formative mini-CEX assessments. Medical Education. 42(1),89–95. 18034797 10.1111/j.1365-2923.2007.02939.x

[ref23] Fokkema J. P. Teunissen P. W. Westerman M. van der Lee N. van der Vleuten C. P. Scherpbier A. J., . . . Scheele F. (2013). Exploration of perceived effects of innovations in postgraduate medical education. Medical Education. 47(3),271–281. 10.1111/medu.12081 23398013

[ref24] French J. C. Colbert C. Y. Pien L. C. Dannefer E. F. & Taylor C. A. (2015). Targeted feedback in the milestones era: Utilization of the ask-tell-ask feedback model to promote reflection and self-assessment. Journal of Surgical Education. 72(6),e274–9. 10.1016/j.jsurg.2015.05.016 26123726

[ref25] Govaerts M. & van der Vleuten C. P. (2013). Validity in work-based assessment: Expanding our horizons. Medical Education. 47(12),1164–1174. 10.1111/medu.12289 24206150

[ref26] Greenhalgh T. Robert G. Macfarlane F. Bate P. & Kyriakidou O. (2004). Diffusion of innovations in service organizations: Systematic review and recommendations. The Milbank Quarterly. 82(4),581–629. 10.1111/j.0887-378X.2004.00325.x 15595944 PMC2690184

[ref27] Harrison C. J. Konings K. D. Schuwirth L. Wass V. & van der Vleuten C. (2015). Barriers to the uptake and use of feedback in the context of summative assessment. Advances in Health Sciences Education: Theory and Practice. 20(1),229–245. 10.1007/s10459-014-9524-6 24906462

[ref28] Heeneman S. Oudkerk Pool A. Schuwirth L. W. van der Vleuten C. P. & Driessen E. W. (2015). The impact of programmatic assessment on student learning: Theory versus practice. Medical Education. 49(5),487–498. 10.1111/medu.12645 25924124

[ref29] Holmboe E. S. (2015). Realizing the promise of competency-based medical education. Academic Medicine: Journal of the Association of American Medical Colleges. 90(4),411–413. 10.1097/ACM.0000000000000515 25295967

[ref30] Holmboe E. S. & Hawkins R. E. (2008). Practical guide to the evaluation of clinical competence. Philadelphia, PA: Mosby,Inc.

[ref31] Holmboe E. S. Yepes M. Williams F. & Huot S. J. (2004). Feedback and the mini clinical evaluation exercise. Journal of General Internal Medicine. 19(5 Pt 2),558–561. 10.1111/j.1525-1497.2004.30134.x 15109324 PMC1492325

[ref32] Kassebaum D. G. & Eaglen R. H. (1999). Shortcomings in the evaluation of students’ clinical skills and behaviors in medical school. Academic Medicine: Journal of the Association of American Medical Colleges. 74(7),842–849. 10.1097/00001888-199907000-00020 10429595

[ref33] Kogan J. R. Holmboe E. S. & Hauer K. E. (2009). Tools for direct observation and assessment of clinical skills of medical trainees: A systematic review. Jama. 302(12),1316–1326. 10.1001/jama.2009.1365 19773567

[ref34] Kogan J. R. Bellini L. M. & Shea J. A. (2003). Feasibility, reliability, and validity of the mini-clinical evaluation exercise (mCEX) in a medicine core clerkship. Academic Medicine: Journal of the Association of American Medical Colleges. 78(10 Suppl),S33–5. 10.1097/00001888-200310001-00011 14557089

[ref35] Kruger R. (1997). Analysing and reporting focus group results. Thousand Oaks,CA: Sage Publications.

[ref36] Malhotra S. Hatala R. & Courneya C. A. (2008). Internal medicine residents’ perceptions of the mini-clinical evaluation exercise. Medical Teacher. 30(4),414–419. 10.1080/01421590801946962 18569664

[ref37] Miller G. E. (1990). The assessment of clinical skills/competence/performance. Academic Medicine: Journal of the Association of American Medical Colleges. 65(9 Suppl),S63–7. 10.1097/00001888-199009000-00045 2400509

[ref38] Norcini J. & Burch V. (2007). Workplace-based assessment as an educational tool: AMEE guide no. 31. Medical Teacher. 29(9),855–871. 10.1080/01421590701775453 18158655

[ref39] Norcini J. J. Blank L. L. Duffy F. D. & Fortna G. S. (2003). The mini-CEX: A method for assessing clinical skills. Annals of Internal Medicine. 138(6),476–481. 10.7326/0003-4819-138-6-200303180-00012 12639081

[ref40] Parker A. & Tritter J. (2006). Focus group method and methodology: Current practice and recent debate. International Journal of Research & Method in Education. 29(1),23–37. 10.1080/01406720500537304

[ref41] Ramani S. & Krackov S. K. (2012). Twelve tips for giving feedback effectively in the clinical environment. Medical Teacher. 34(10),787–791. 10.3109/0142159X.2012.684916 22730899

[ref42] Sargeant J. McNaughton E. Mercer S. Murphy D. Sullivan P. & Bruce D. A. (2011). Providing feedback: Exploring a model (emotion, content, outcomes) for facilitating multisource feedback. Medical Teacher. 33(9),744–749. 10.3109/0142159X.2011.577287 21854151

[ref43] Schuwirth L. & Ash J. (2013). Assessing tomorrow’s learners: In competency-based education only a radically different holistic method of assessment will work. Six things we could forget. Medical Teacher. 35(7),555–559. 10.3109/0142159X.2013.787140 23641916

[ref44] Schuwirth L. W. & Van der Vleuten C. P. (2011). Programmatic assessment: From assessment of learning to assessment for learning. Medical Teacher. 33(6),478–485. 10.3109/0142159X.2011.565828 21609177

[ref45] Stalmeijer R. E. Mcnaughton N. & Van Mook W. N. (2014). Using focus groups in medical education research: AMEE guide no. 91. Medical Teacher. 36(11),923–939. 10.3109/0142159X.2014.917165 25072306

[ref46] Van de Wiel M. W. Van den Bossche P. Janssen S. & Jossberger H. (2011). Exploring deliberate practice in medicine: How do physicians learn in the workplace? Advances in Health Sciences Education: Theory and Practice. 16(1),81–95. 10.1007/s10459-010-9246-3 20848187 PMC3074057

[ref47] Van der Vleuten C. P. Schuwirth L. W. Driessen E. W. Dijkstra J. Tigelaar D. Baartman L. K. & van Tartwijk J. (2012). A model for programmatic assessment fit for purpose. Medical Teacher. 34(3),205–214. 10.3109/0142159X.2012.652239 22364452

[ref48] Van Rossum T. R. Scheele F. Scherpbier A. J. Sluiter H. E. & Heyligers I. C. (2016). Dealing with the complex dynamics of teaching hospitals. BMC Medical Education. 16(1), 104-016-0623-3. 10.1186/s12909-016-0623-3 PMC4822260 27048264

[ref49] Watling C. J. Kenyon C. F. Schulz V. Goldszmidt M. A. Zibrowski E. & Lingard L. (2010). An exploration of faculty perspectives on the in-training evaluation of residents. Academic Medicine: Journal of the Association of American Medical Colleges. 85(7),1157–1162. 10.1097/ACM.0b013e3181e19722 20592512

[ref50] Watling C. J. Kenyon C. F. Zibrowski E. M. Schulz V. Goldszmidt M. A. Singh I. Lingard L. (2008). Rules of engagement: Residents’ perceptions of the in-training evaluation process. Academic Medicine: Journal of the Association of American Medical Colleges. 83(10 Suppl),S97–100. 10.1097/ACM.0b013e318183e78c 18820513

[ref51] Weston P. S. & Smith C. A. (2014). The use of mini-CEX in UK foundation training six years following its introduction: Lessons still to be learned and the benefit of formal teaching regarding its utility. Medical Teacher. 36(2),155–163. 10.3109/0142159X.2013.836267 24099402

