# Supplementary material for: Student Perceptions of Assessment for Workplace Learning and Summative Decisions Using Frequent Mini-CEXs
Source: MedEdPublish (2016). 2017 Jun 22;6:113. [Version 1] doi: 10.15694/mep.2017.000113 (PMC10885268; doi:10.15694/mep.2017.000113)
Supplement: Peer review history

# Reviewer report: Reviewer response for version 1

**DOI:** 10.21956/mep.19266.r28000

**Published:** 2019-03-07

**Authors:** Trevor Gibbs

**Affiliations:** 1 AMEE

This review has been migrated. The reviewer awarded 4 stars out of 5 A well written paper that covers an important area; I greatly appreciated the very useful reference list on the subject. I suppose this paper shows that we are still unsure about some of the workings on workplace-based assessment, especially the mini-CEX. However it does show that in part the students appreciate the activity although it does enlighten the need for faculty / trainer learning in the area.

Reviewer Expertise:

NA

No decision status is available

# Reviewer report: Reviewer response for version 1

**DOI:** 10.21956/mep.19266.r27999

**Published:** 2019-02-18

**Authors:** Ken Masters

**Affiliations:** 1 Sultan Qaboos University

This review has been migrated. The reviewer awarded 4 stars out of 5 A very well written paper indeed. The authors begin with a detailed analysis of the literature, leading to their research questions on the impact of low-stakes min-CEXs’ affect on students’ perception of learning and their relationship with end-of-rotation summative clinical performance evaluations.It is a pity that one of the focus groups was so small, but that can sometimes happen in this kind of research. The questions were appropriately open enough to allow for a wide range of experiences to be identified.Although the study is of value for most readers contemplating Mini-CEXs, the study itself could benefit from a somewhat larger student sample, and, as the authors note, a comparison with study of faculty. Perhaps a later study might be undertaken.Still, overall, a useful paper and a good read.

Reviewer Expertise:

NA

No decision status is available

# Reviewer report: Reviewer response for version 1

**DOI:** 10.21956/mep.19266.r27998

**Published:** 2017-06-26

**Authors:** Tan Nguyen

**Affiliations:** 1 Deakin University

This review has been migrated. The reviewer awarded 3 stars out of 5 This research published provides a good starting point to improve clinical education practice at a local level. I can certainly relate to this study, having been a past student from the Melbourne Dental School with the use of the mini-CEX, which is used routinely for all clinical encounters, and currently as a clinical educator in the same institution. Since my studies, the assessment of the mini-CEX had changed from an overall professional judgement grade to a clinical competency grading according to the skill domains, similar to the domains used in this research. As mentioned, it would be great to investigate how the new type of assessment practice improved learning opportunities as reported by the clinical educators. It would be ideal also to include the students' feedback on the mini-CEX prior to the clinical educators feedback.

Reviewer Expertise:

NA

No decision status is available
